# Processing of Natural Echolocation Sequences in the Inferior Colliculus of Seba’s Fruit Eating Bat, *Carollia perspicillata*

**DOI:** 10.1523/ENEURO.0314-17.2017

**Published:** 2017-12-13

**Authors:** M. Jerome Beetz, Sebastian Kordes, Francisco García-Rosales, Manfred Kössl, Julio C. Hechavarría

**Affiliations:** 1 Institut für Zellbiologie und Neurowissenschaft, Goethe-Universität, Frankfurt am Main 60438, Germany; 2 Department of Behavioral Physiology and Sociobiology, Biozentrum, University of Würzburg, Am Hubland, Würzburg 97074, Germany

**Keywords:** suppression, orientation, bats, acoustic, inferior colliculus, temporal processing

## Abstract

For the purpose of orientation, echolocating bats emit highly repetitive and spatially directed sonar calls. Echoes arising from call reflections are used to create an acoustic image of the environment. The inferior colliculus (IC) represents an important auditory stage for initial processing of echolocation signals. The present study addresses the following questions: (1) how does the temporal context of an echolocation sequence mimicking an approach flight of an animal affect neuronal processing of distance information to echo delays? (2) how does the IC process complex echolocation sequences containing echo information from multiple objects (multiobject sequence)? Here, we conducted neurophysiological recordings from the IC of ketamine-anaesthetized bats of the species *Carollia perspicillata* and compared the results from the IC with the ones from the auditory cortex (AC). Neuronal responses to an echolocation sequence was suppressed when compared to the responses to temporally isolated and randomized segments of the sequence. The neuronal suppression was weaker in the IC than in the AC. In contrast to the cortex, the time course of the acoustic events is reflected by IC activity. In the IC, suppression sharpens the neuronal tuning to specific call-echo elements and increases the signal-to-noise ratio in the units’ responses. When presenting multiple-object sequences, despite collicular suppression, the neurons responded to each object-specific echo. The latter allows parallel processing of multiple echolocation streams at the IC level. Altogether, our data suggests that temporally-precise neuronal responses in the IC could allow fast and parallel processing of multiple acoustic streams.

## Visual Abstract

**Figure:**
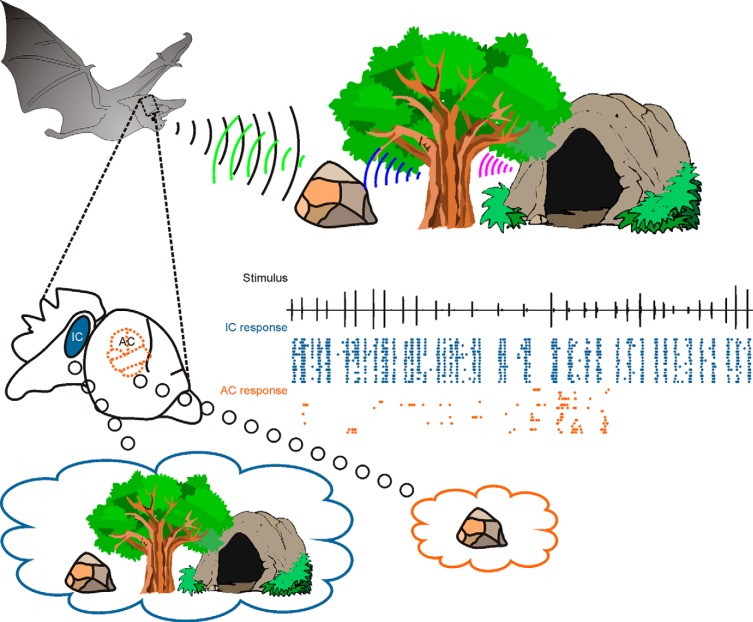

## Significance Statement

High stimulus rates usually result in a reduction of neuronal responses that can be described as suppression or adaptation. It remains unclear how neuronal suppression influences sensory processing in animals that rely on high stimulus rates, as it is the case of bats. The present study investigates how natural echolocation sequences are processed in the bat’s inferior colliculus (IC). We report that collicular suppression enhances the signal-to-noise ratio of the spiking activity without degrading the temporal processing of echolocation sequences. Collicular suppression allows for a high tracking ability of the stimulus envelope and for the parallel processing of multiple auditory streams.

## Introduction

The sensory world is dynamic and animals continuously receive sensory information from the environment. The temporal context, in which stimuli occur often carries behaviorally relevant information. Temporal parameters, like repetition rate, signal duration, or inter-signal intervals, are used to identify conspecifics, a strategy that has been described in *Drosophila* (Coen et al., 2014), crickets (Ronacher et al., 2015; Hedwig, 2006), and frogs (Feng et al., 1990; Gerhardt, 2005). Bats also rely on fast acoustic repetition rates for coping in everyday life scenarios. They orientate acoustically in the dark using echolocation by integrating high acoustic rates of call-echo information (Moss and Surlykke, 2010; Simmons, 2012; Kössl et al., 2015). Although fast acoustic repetition rates are important for many animal species, encoding these fast time-varying signals is challenged by the fact that repetitive stimuli often degrade temporal processing along the auditory pathway by evoking neuronal suppression from the auditory nerve on (Harris and Dallos, 1979; Wiggs and Martin, 1998; Joris et al., 2004; Grill-Spector et al., 2006).

To unravel fundamental principles of temporal processing, it is important to stimulate animals with ethologically relevant stimuli in a natural temporal context (Margoliash and Fortune, 1992; Carruthers et al., 2013; Woolley and Portfors, 2013; Theunissen and Elie, 2014; Beetz et al., 2016a). The present study tested, neuronal processing of natural sound sequences, with special focus on the relevance of a natural temporal context, in the inferior colliculus (IC) of the frugivorous bat *Carollia perspicillata*. The IC is considered an important structure for the processing of temporal sound attributes. Collicular neurons are often selective to stimulus parameters such as interaural intensity and time differences (Klug et al., 1995), sound duration (Casseday et al., 1994), frequency modulation (Casseday et al., 1997), amplitude modulation (Borina et al., 2008), as well as spectral and temporal sound combinations (combination sensitive neurons; Wenstrup et al., 2012). The increased neuronal selectivity in collicular neurons, compared to the rather unselective neuronal responses of the auditory brainstem, makes the IC an important center for the extraction and integration of sensory stimuli features (Casseday and Covey, 1996; Wenstrup et al., 2012).

We used bats as a model to study the processing of sound sequences, because these animals have to cope with fast time-varying acoustic streams in everyday situations. During echolocation, bats emit high-frequency biosonar calls in a repetitive manner. The calls reflect off surrounding objects resulting in echoes. Bats use echoes to detect, localize, and identify objects thus creating an acoustic image of the surrounding (Neuweiler, 1990; Moss and Surlykke, 2010; Kössl et al., 2014; Wohlgemuth et al., 2016). They infer the distance to objects from the echo delay, which represents the time interval between call emission and echo arrival (Hartridge, 1945; Simmons, 1973). Neurons involved in distance processing respond selectively to call echo pairs, in which the echo follows the call with a certain delay (Grinnell, 1963a; Feng et al., 1978; Suga et al., 1978; Suga and O'Neill, 1979; Hagemann et al., 2010).

Neurophysiological studies in bats revealed that the stimulus repetition rate affects neuronal tuning. Neurons become more selectively tuned to sound duration (Zhou and Jen, 2006), sound frequency (Jen et al., 2001; Smalling et al., 2001), echo delay (O'Neill and Suga, 1982; Wong et al., 1992; Bartenstein et al., 2014), amplitude (Galazyuk et al., 2000), and azimuthal position (Wu and Jen, 1996), when the stimuli are presented at high rates. The aforementioned studies investigated the effect of the stimulus rate on the neuronal tuning by stimulating bats with echolocation sequences composed of constant inter-call interval. However, in real life scenarios, physical parameters like inter-call interval, call duration and the spectral composition of the calls vary during an echolocation sequence (Griffin, 1953; Neuweiler, 1990). Thus, to understand the neuroethological roles of the auditory centers involved in processing echolocation signals, it is necessary to investigate neuronal processing with natural echolocation sequences. So far, processing of natural echolocation sequences has been characterized in the superior colliculus of the insectivorous bat *Eptesicus fuscus* (Wohlgemuth and Moss, 2016) and in the auditory cortex (AC) of *C. perspicillata* (Beetz et al., 2016a,b). Cortical results have shown that the natural temporal context evokes neuronal suppression which results into a high neuronal selectivity to particular call echo pairs (Beetz et al., 2016a) or to object-specific echo information (Beetz et al., 2016b). However, it remains largely unknown whether the response of subcortical neurons displays a sharper echo-delay selectivity when studied with natural echolocation sequences.

## Materials and Methods

### Animals

Electrophysiological recordings from the IC were performed in six adult females of the frugivorous bat *C. perspicillata*. Bats were taken from a breeding colony at the Institute for Cell Biology and Neuroscience (Goethe-University). The animal use in this study complies with all current German laws on animal experimentation and it is in accordance with the Declaration of Helsinki. All experimental protocols were approved by the Regierungspräsidium Darmstadt (experimental permit #F104/57).

### Acoustic stimuli

Frequency-level receptive fields were calculated from neuronal responses to pure tones of 10-ms duration (0.5-ms rise-fall time) whose frequency and intensity was varied. Sound frequencies ranged from 5–95 kHz (5-kHz steps) and the sound pressure levels were between 30- and 90-dB SPL (10-dB steps). Sound levels were adjusted based on the speaker’s calibration curve. Each frequency-level combination was randomly presented five times with a 400-ms interstimulus interval.

Natural echolocation sequences were recorded in a pendulum (Henson et al., 1982; Beetz et al., 2016a,b). The bat was placed in a pendulum and swung toward different objects. During the swing, the animal broadcasts echolocation calls. The calls and echoes, arising from call reflections from the surrounding objects, were recorded with an ultrasound sensitive microphone (CM16/CMPA, Avisoft Bioacoustics). The microphone was attached to the pendulum and positioned medially above the animal’s head. The distance between the animals’ ears and the microphone membrane was set to 4 cm. The microphone had a sensitivity of 50 mV/Pa and an input-referred self-noise level of 18-dB SPL. Sound signals were acquired with an UltraSoundGate 116 Hm mobile recording interface (Avisoft Bioacoustics, RRID: SCR_014436) and a sampling rate of 375 kHz (16-bit precision).

**Figure 1.**
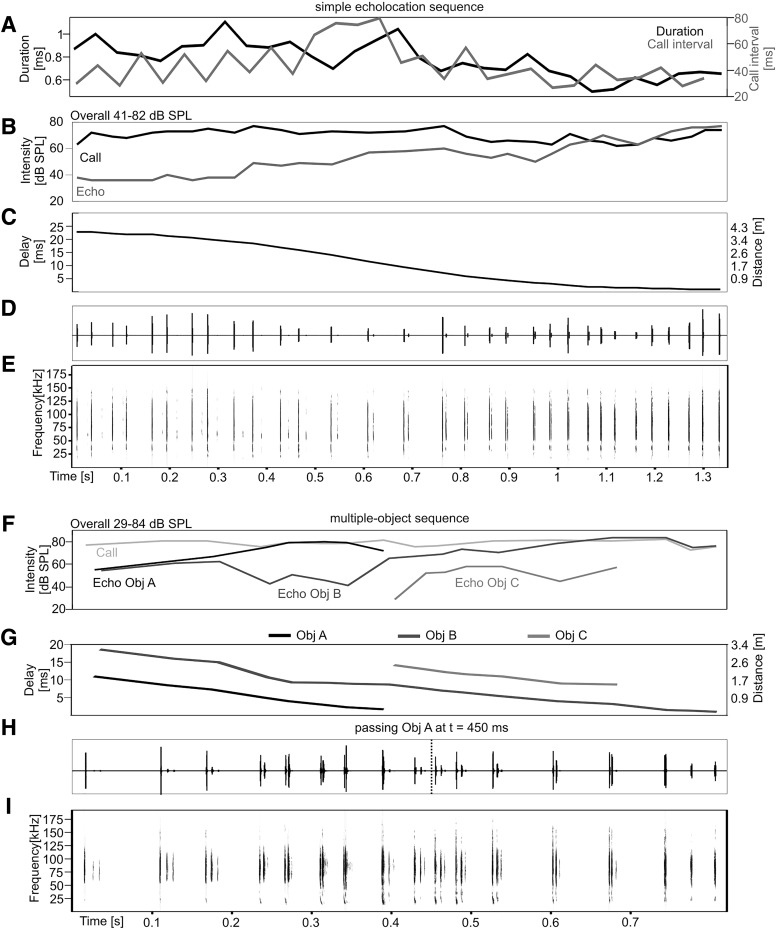
Natural echolocation sequences used as acoustic stimuli. Two representative echolocation sequences recorded during a forward swing of the pendulum. ***A–E***, Energetic, spectral, and temporal parameters characterizing the simple echolocation sequence. The sequence contains echo information from one object (acrylic glass wall positioned at the end of the swing). ***A***, Call duration (black trace) and call interval (gray trace) over the time course. Call durations and intervals decrease toward the end of the swing. ***B***, Call intensity is independent from object distance and varies between 67 and 82-dB SPL. Echo intensity increases during the approach from 41- to 82-dB SPL. ***C***, Echo delays decrease over time. Oscillogram (***D***) and spectrogram (***E***) of the simple echolocation sequence. ***F–H***, Same plots as in ***B–E*** but with physical parameters from the multiple-object sequence. During the swing the bat faced three objects. Thus, each call was followed by at least two echoes coming from different objects. Object A is overflown by the animal between 400 and 450 ms. Therefore, echolocation signals after 450 ms do not contain echo information from object A.

For the present study, two representative echolocation sequences recorded in the pendulum were used as acoustic stimuli for electrophysiological recordings. Both sequences were recorded during the forward swing of the pendulum. The pendulum swung at an average speed of 3 m/s (speed calculated as the total *x*-axis displacement/time). Note that during an approach flight the velocity of *C. perspicillata* ranges between 2 and 3 m/s (Thies et al., 1998). For the first sequence (Fig. 1*A–E*, simple echolocation sequence), the bat was swung toward an acrylic glass wall (depth: 0.3 cm; width 50 cm; height: 150 cm). Each echolocation call was reflected once at the acrylic wall. Thus, each call was followed by an echo with a distance-dependent time delay (defined as echo delay). Echo delays decreased from 22.8 to 1.1 ms, which correspond to distance changes from 3.9 to 0.17 m (Fig. 1*C*). Echolocation sequence parameters fell within the natural range of *C. perspicillata* (Table 1; Thies et al., 1998). Consistent with findings in freely flying bats (Thies et al., 1998), the call duration decreases as the bat approaches the object in the pendulum paradigm (Fig. 1*A*).

**Table 1.** Temporal call parameters of the echolocation sequences, used in the present study, are compared with call parameters measured in the field (from Thies et al., 1998**)**

	Call duration (ms)	Call interval (ms)	Duty cycle (%)
Simple echolocation sequence	0.79 ± 0.15	40 ± 14.85	2 ± 0.56
Multiobject sequence	1.29 ± 0.25	49.33 ± 20.9	3.06 ± 1.14
Freely flying (from Thies et al., 1998)	0.8 ± 0.2	42.2 ± 25.8	2.4 ± 1.1

For recording a multiple-object sequence (Fig. 1*F–I*), three objects were positioned along the swing trajectory. Object A was a dummy rock (depth: 65 cm; width 95 cm; height: 35 cm) made of papier-mâché and it was overflown by the animal at time point *t* = 450 ms (Fig. 1*H*, dashed vertical line). Object B, a wooden plate (depth: 0.8 cm; width: 21 cm; height: 21 cm) was positioned 130 cm after object A and 20 cm in front of object C. Object C was the acrylic glass wall from the simple echolocation sequence. The swing of the pendulum stopped directly in front of object B. The objects were positioned so that each echolocation call was followed by at least two echoes. One echolocation call was followed by echoes from all three objects (i.e., call #8 at *t* = 0.39 s; Fig. 1*H*). The recorded echolocation sequences were resampled from 375 kHz to 384 kHz. The “noise reduction” function (FFT length 256; precision 16) of the software Avisoft SAS Lab Pro (Avisoft Bioacoustics, RRID: SCR_014438) filtered background noise in the frequency domain. The spectro-temporal structure of call and echoes were not affected due to the high signal-to-noise ratio of the recording. An elliptic filter (order 8) in the software BatSound (Pettersson Elektronik AB) eliminated the remaining sound artifacts from background noise.

For investigating the relevance of the stimulus history, the simple echolocation sequence was cut into segments using a custom-written Matlab script (R2009, RRID: SCR_001622). Each segment contained a call and an echo. In the rest of the manuscript, we will refer to the segment as “call-echo elements.” The call-echo elements were randomly presented with a 400-ms interstimulus time interval, from here on called “element situation.” The neuronal response to the element situation was compared with the response elicited by the natural echolocation sequence, from here on called “sequence situation.”

The multiple-object sequence was transformed into single-object sequences by manually deleting object-specific echoes in the software BatSound (PettersonElektronik AB). According to the distances to objects, the echoes from object A and B should be separated by 7.65 ms. Echoes from object B and object C should be separated by 1.2 ms. Calculations are based on the equation:

$$R=D\times \frac{c}{2}$$

*R* represents the distance, *D* the echo delay and *c* the sound velocity in air at 20°C. In our multiple-object sequence, echoes from object A and object B were separated by 6.7 ± 0.9 ms and echoes from object B and object C by 5.3 ± 0.2 ms. The discrepancy of the echo delays between the echoes of object B and object C might be because echoes from object C could derive from the off-axis of the sonar beam. Thus, echoes from object C had to cover longer distances than echoes from object B. Echoes from different objects did not temporally overlap, which allowed us to delete object-specific echoes.

For stimulation, acoustic signals were played using an Exasound E18 sound card (ExaSound Audio Design) at a sampling rate of 384 kHz. To avoid sound artifacts, such as clicks during stimulation, the acoustic stimuli were multiplied by a fading function resulting in smooth rise-fall times of 0.5 ms. The acoustic stimuli were transferred to an audio amplifier (Rotel power amplifier, RB_850_), before they were played through a calibrated speaker (ScanSpeak Revelator R2904/7000, Avisoft Bioacoustics). The speaker was located 15 cm from the bat’s right ear. Speaker calibration was done with a ¼-inch microphone (Brüel & Kjaer, model 4939) connected to a custom-made microphone amplifier.

While recording neuronal signals from the left IC, the sequence situation, the element situation, the multiple-object sequence, and the multiple-object sequences with deleted echoes were presented 15 times with an interstimulus interval of 400 ms to an anaesthetized bat. Sound pressure levels of calls and echoes are plotted in Figure 1*B*,*F* for the simple echolocation sequence and multiple-object sequence, respectively.

### Electrophysiological recordings

For anesthesia, bats were subcutaneously injected with a mixture of ketamine (10 mg/kg^−1^ Ketavet, Pharmacia GmbH) and xylazine (38 mg/kg^−1^ Rompun, Bayer Vital GmbH). A local anesthetic (xylocaine 2%, AstraZeneca GmbH) was applied topically onto the skin of the bat’s head. A longitudinal midline cut was made through the skin. Skin and muscles covering the skull were removed. For fixating the bat’s head during the recordings, a custom-made metal rod (1-2 cm in length, 0.1 cm in diameter) was glued with acrylic glue (Heraeus Kulzer GmbH), super glue (UHU), and dental cement (Paladur, Heraeus Kulzer GmbH) at the rostral end of the skull. After two recovery days from surgery, a craniotomy covering an area of 1 mm^2^ above the midbrain was done to gain access to the left IC.

Electrophysiological recordings were conducted in a sound-proofed and electrically-shielded chamber. During anesthesia, the temperature of the bat holder was kept constant at 37°C with a heating pad positioned below the immobile bat. Neuronal recordings were performed using single glass electrodes (resistance 4–10 MΩ when filled with 3 Mol KCl) which were constructed by pulling borosilicate capillaries (GB120F-10, Science Products) with a Flaming/Brown horizontal puller (P97, Sutter). Glass electrodes were positioned 2-3 mm lateral from the midline of the scalp. A prominent blood vessel running dorsally over the rostral cerebellum was used as landmark for determining the rostro-caudal position of the IC. The electrode was penetrated orthogonally to the brain surface, through an intact dura mater. Recording depths were measured with a Piezo Manipulator (PM 10/1, Science products GmbH). The brain surface was used as reference point (0 µm) for depth measurement and the recording depths ranged from 610 up to 6210 µm. A silver wire, placed 1-2 cm rostral from the recording electrode and touching the brain surface of nonauditory areas, was used as grounding electrode. Neuronal data acquisition was performed using a wireless multichannel recording system (Multi Channel Systems MCS GmbH), at a sampling rate of 20 kHz (per channel) and 16-bit precision. One channel of the multichannel recording system was connected to the recording electrode while the remaining channels were short-circuited and connected to ground. One recording session lasted on average 4 h. Recordings were performed chronically in each animal. After each recording session, the animal had at least one day for recovery. The health status of the animal was documented with health reports, including daily weight measurements.

### Analysis of neuronal recordings

Spike events were detected with a multiunit-specific threshold that was based on the spike amplitude. For each multiunit, spike threshold was kept constant throughout the stimulation protocol, thus ensuring that the same multiunit activity was recorded for each stimulus. Spike detection was based on spike amplitude relative to recording noise level. The spikes were sorted based on the first three principle components of the spike waveforms and they were clustered automatically using the KlustaKwik algorithm (Lewicki, 1998, RRID: SCR_014480). Only the cluster with the largest number of spikes was used for further analysis. Neuronal responses from the IC were analyzed in 90 spike-sorted single units.

Initially, the characteristic frequency (CF), which represents the frequency to which the neuron is most sensitive, was calculated for each unit. Neuronal responses to the echolocation sequences were assessed from units with CFs higher than 35 kHz (*n* = 79). Neuronal data from 149 cortical units from a previous study (Beetz et al., 2016a) were used and compared to the IC data.

A suppression rate calculated with the following equation was calculated for each unit:

$$suppression\;rate=1-\frac{\#\;spikes(sequence\;situation)}{\#\;spikes(element\;situation)}$$

Unless otherwise mentioned, IC data were analyzed with poststimulus time histograms (PSTHs) with a binsize of 2 ms. The tracking ability of the units was assessed by cross correlating the PSTHs with the down-sampled envelope of the stimulus energy. Note that the PSTH binsize used for the cross-correlation (CC) was 1 ms for collicular and cortical units.

The calculation of the signal-to-noise-ratio was based on normalized PSTHs. PSTHs were normalized in a unit-specific manner, relative to each unit’s maximum spike count per bin when considering both the element and sequence situations. To distinguish between stimulus evoked responses (signal) and background activity (noise), a threshold was set to 50% of the maximum value of the normalized PSTHs. Bins crossing that threshold were defined as the “signal” and compared to the remaining bins that represent the “noise.” The signal-to-noise ratio of a call-echo element represents the sum of the number of spikes in bins defined as signals divided by the total number of spikes elicited by that call-echo element. Thus, a signal-to-noise ratio of 1 indicates that each spike elicited in response to the call-echo element, was assigned to a signal and that the noise level was zero. A signal-to-noise ratio was quantified for the responses to each call-echo element. For obtaining a unit specific signal-to-noise ratio, we calculated the median values of the signal-to-noise ratios calculated in response to each call-echo element.

Because of the variable inter-call intervals of the echolocation sequence, conventional PSTHs with constant bin-sizes could not be used to assess the tuning of the collicular units to specific call-echo elements. Therefore, “activity histograms” were calculated by assigning each spike according to its relative time point to the preceding call-echo element. In other words, activity histograms represent PSTHs with variable binsizes that correspond to the time window of the call-echo elements.

To describe delay tuning, two different parameters were calculated. The best delay represents the echo delay (represented by call-echo elements) that elicits the strongest response. The median delay was calculated by measuring the median time point of the evoked spikes. The median time point was then assigned to a call-echo element. The echo delay, encoded by the call-echo element, represents the median delay. In contrast to the best delay calculation, the median delay calculation considers each elicited spike. Data analysis was done in Matlab 2014 (MathWorks), and statistics in GraphPad Prism 5 (GraphPad Software; *p < 0.05; **p < 0.01; ***p < 0.0001, RRID: SCR_002798).

## Results

### Tonotopy

**Figure 2.**
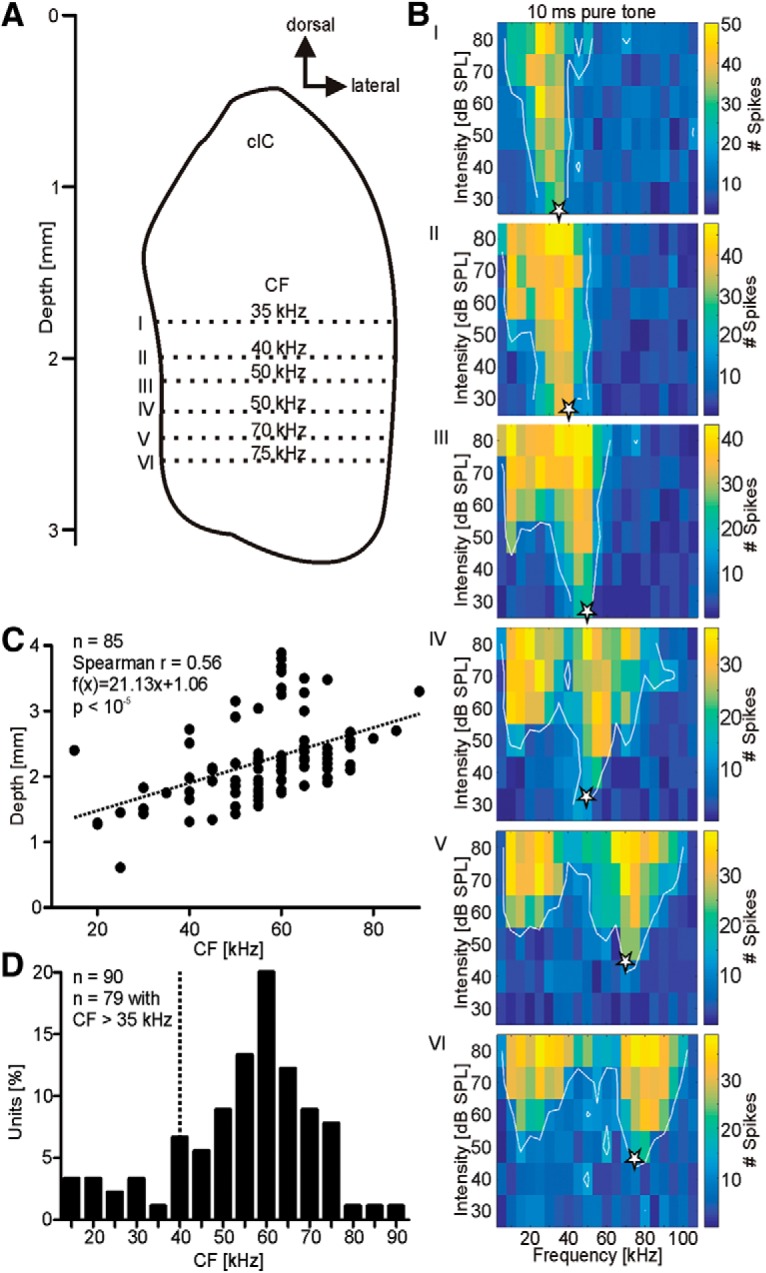
Tonotopy of the cIC in *C. perspicillata*. ***A***, Schematic frontal view on the cIC. CFs increased with recording depth. ***B***, Representative frequency receptive fields from six units recorded from different depths of one penetration track. Depths are indicated by roman numerals (I-VI; ***A***). The CFs of the units are indicated by white stars in the receptive fields and increase with the recording depths. High-frequency tuned neurons typically had multipeaked frequency receptive fields. ***C***, Scatter plot shows the increase of the CF along the recoding depth for 85 collicular units. ***D***, Histrogram represents the distribution of CFs from 90 collicular units recorded in the present study. Units with CFs higher than 35 kHz (dashed vertical line) were classified as high-frequency tuned units and were tested further with the echolocation sequences from Figure 1.

Extracellular recordings were obtained from 90 auditory sensitive and spike-sorted single units from the central nucleus of the IC (cIC) of *C. perspicillata*. Recordings were from the cIC because a clear tonotopy was found along the dorso-ventral axis, which is characteristic for the cIC (Fig. 2; Grinnell, 1963b; Pinheiro et al., 1991; Schmidt et al., 1991; Sterbing et al., 1994; Jen and Chen, 1998). We determined each unit’s CF based on its frequency receptive field (Fig. 2*B*). The CF represents the frequency to which the unit is most sensitive (Fig. 2*B*, white stars). Recording depths were calculated for 85 units and plotted against the CF (Fig. 2*C*). A Spearman CC analysis and linear regression depict that the CF increased with increasing recording depth [*R* = 0.56, f(x) = 21.13x + 1.06; *p* < 10^−5^]. Note that the neurons show a multipeaked receptive field with increasing depth (Fig. 2*B*). Thus, high-frequency tuned neurons of the IC receive excitatory input from low (<35 kHz) and high frequencies (>35 kHz). Multipeaked receptive fields have been described for a relatively lower number of neurons in the IC of the mustached bat (Holmstrom et al., 2007), *E. fuscus* (Casseday and Covey, 1992), and *Myotis oxygnathus* (Vasil`ev and Matyushkin, 1967). In the AC of *C. perspicillata*, high-frequency tuned neurons are also multipeaked in *C. perspicillata* (Hagemann et al., 2010; Hagemann et al., 2011).

In comparison to communication signals, echolocation calls have their main energy at high frequencies (Hechavarría et al., 2016a). Therefore, we tested neuronal responses to natural echolocation sequences (see sequences in Fig. 1) only in neurons with CFs higher than 35 kHz (Fig. 2*D*, 79 units positioned to the right of the dashed line). The remaining eleven units were defined as low frequency tuned neurons and were not taken into consideration for the remaining analysis.

### Suppression at IC level is weaker than at the cortical level

**Figure 3.**
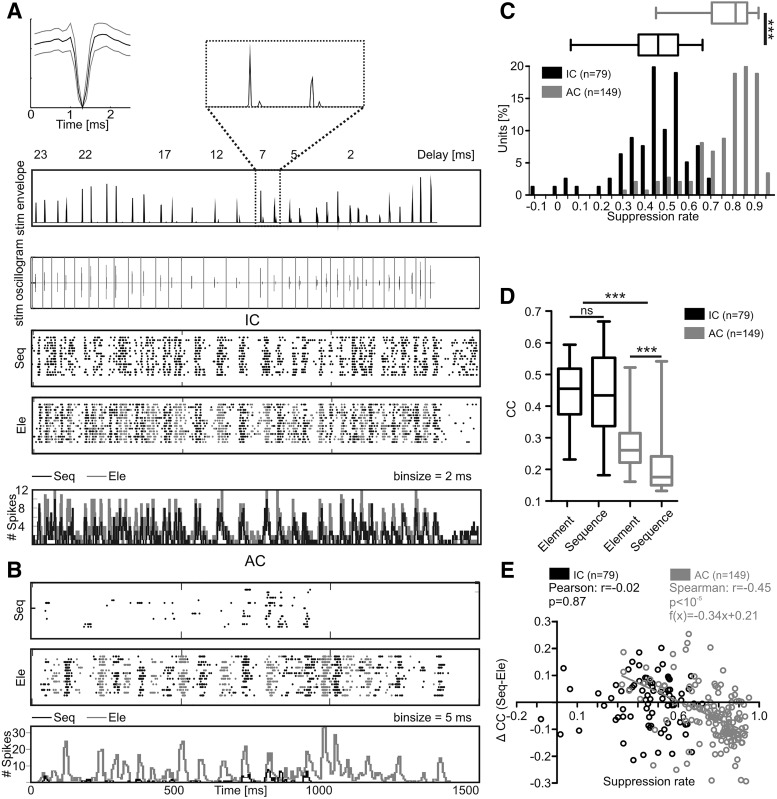
Collicular neurons synchronize more their discharges to the stimulus envelope than cortical neurons. ***A***, Neuronal response from a representative unit of the IC. Median (black trace), 25th, and 75th quantile (gray traces) spike wave form is shown in the left upper corner. Stimulus envelope and oscillogram of the stimulus are shown below. Two call-echo elements of the stimulus envelope are magnified on top. Neuronal responses are shown as raster plots, where one dot indicates an action potential, and as PSTHs. In the sequence situation (Seq), the animals were stimulated with the natural echolocation sequence. In the element situation (Ele) single call-echo elements of the sequence were randomly presented with a 400-ms interstimulus time interval. The time borders of the call-echo elements are indicated by gray vertical lines in the oscillogram. For visualization, the raster plot of the element situation was created by concatenating the neuronal responses to the call-echo elements. Alternating gray scales visualize which action potentials were evoked by which call-echo element. ***B***, Neuronal response from a unit of the AC to the sequence and element situation. Raster plots and PSTHs are organized as in ***A***, except that the binsize of the PSTH was adjusted to 5 ms. ***C***, Boxplot and histogram of the suppression rates in the IC and AC. In the sequence situation, IC units were less suppressed than AC units (Mann–Whitney *t* test: *p* < 10^−5^). ***D***, Boxplots showing the CC values calculated between PSTHs, with a binsize of 1 ms, and the stimulus envelope. In the IC (black boxplots), the CC values did not differ between the element and sequence situation (*p* > 0.05), indicating that subcortical suppression prevails the neuronal synchronization to the stimulus envelope. CC values from the AC (gray boxplots) were significantly smaller than in the IC (*p* < 10^−5^) and decreased further from the element to the sequence situation (*p* < 10^−5^). Wilcoxon signed rank test for testing between stimulus conditions and Kruskal Wallis one-way ANOVA and Dunns multiple comparison *post hoc* test for comparing between IC and AC. ***E***, Scatter plot shows that in AC (gray circles) the suppression rate was correlated with the decrease of neuronal synchronization from the element to the sequence situation (Spearman: *r* = −0.45; *p* < 10^−5^, f(x) = −0.34x + 0.21). No correlation between the suppression rate and changes in neuronal synchronization was found in the IC (black circles; Pearson: *r* = −0.02; *p* = 0.87).

We analyzed neuronal responses from 79 collicular units that were recorded while the bats were listening to an echolocation sequence [sequence situation; black raster and PSTH (binsize = 2 ms) in Fig. 3*A*]. The sequence mimicked a stimulus scenario that the bat could perceive when flying toward an object. To quantify the influence of the temporal context of the sequence on the neuronal response, the bats were also stimulated with the temporally isolated call-echo elements of the sequence. Temporal isolation means that the call-echo elements were randomly presented with a 400-ms interelement interval (element situation; black and gray raster and gray PSTH in Fig. 3*A*). In comparison to the results from the cortex (Fig. 3*B*), the responses of IC units were less suppressed in the sequence situation (Fig. 3*A*) when compared to the element situation. Neuronal data from 79 collicular and 149 cortical units (database of cortical units based on Beetz et al., 2016a) demonstrate that the suppression is significantly weaker in the IC than in the cortex (Fig. 3*C*; median: 0.46 and 0.81 for IC and cortex, respectively; Mann–Whitney *t* test: *p* < 10^−5^). The suppression was quantified based on a suppression rate that is calculated as the ratio of evoked spikes in the sequence and element situations, and by subtracting that ratio from 1 (for details see Materials and Methods). Note that the IC was not free of suppression which is reflected by suppression rates that differed significantly from 0 (Sign test: *p* = 2.6 × 10^−22^).

The time course of the echolocation sequence was more accurately represented by IC than AC neurons (Fig. 3*A*,*B*, example raster plots). To evaluate the neuronal synchronization to the acoustic events of the echolocation sequence in the sequence situation, the PSTHs (binsize = 1 ms) in response to the element and sequence situation were cross-correlated with the stimulus envelope. High CC values indicate a high neuronal synchronization and a high tracking ability of the neurons. In the IC, the CC values did not differ significantly between the element and sequence situation indicating that suppression at the IC did not affect the neuronal synchronization (Fig. 3*D*; median: 0.45 and 0.43 for element and sequence situation, respectively; Wilcoxon signed rank test: *p* = 0.86). In contrast to the IC, cortical neurons less synchronized their discharges to the stimulus envelope. CC values calculated for cortical neurons were significantly lower than the CC values from IC neurons (median: 0.45 and 0.43 for IC and 0.26 and 0.18 for cortex; Kruskal Wallis one-way ANOVA and Dunns multiple comparison *post hoc* test: *p* < 10^−5^). For the cortical neurons, CC values were significantly higher in the element than in the sequence situation (Wilcoxon signed rank test: *p* < 10^−5^). The degradation of neuronal synchrony was based on the cortical suppression, which is indicated by a negative correlation between suppression rate and CC values [Fig. 3*E*; Spearman: *r* = −0.45; *p* < 10^−5^; f(x) = −0.34x + 0.21]. At the level of the IC, no correlation was found between the suppression rate and tracking ability (Pearson: *r* = −0.02; *p* = 0.87).

### Collicular suppression increases signal-to-noise ratio

**Figure 4.**
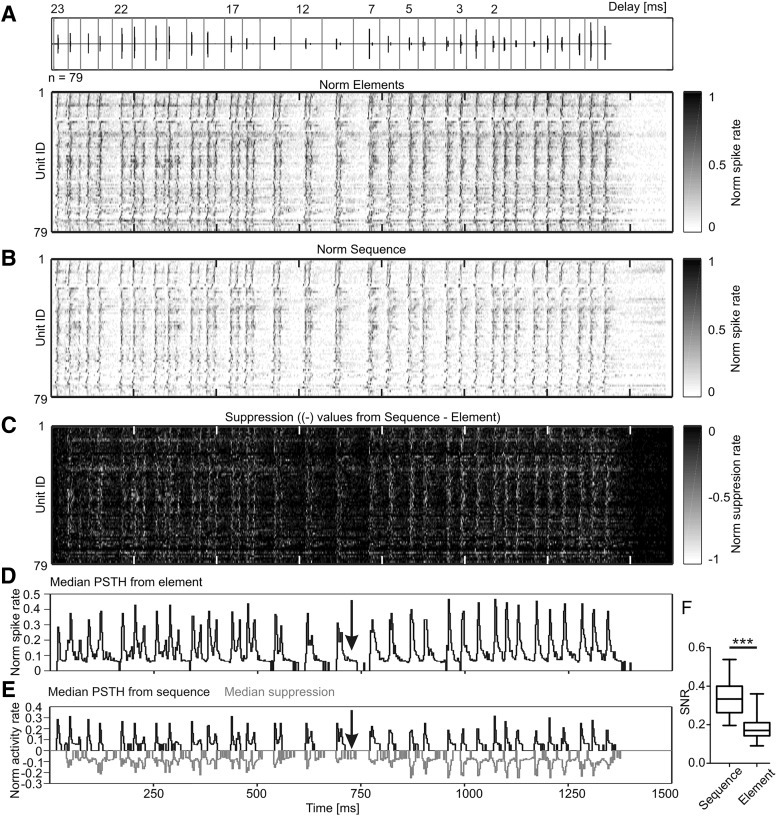
Collicular suppression increases signal-to-noise ratio. ***A***, top, Oscillogram of the stimulus. Vertical gray lines define the time borders of the call-echo elements. Bottom, Population activity map in response to the element situation. Normalized PSTHs were transformed into grayscale coded activity maps. Neuronal activity from each unit is represented in a single row. ***B***, Population activity map in response to the sequence situation. ***C***, Population suppression map calculated by subtracting population activity map in response to the element situation (***A***) from the map calculated in response to the sequence situation (***B***). Respectively, bright and dark bins represent high and weak suppression rates. ***D***, ***E***, Median PSTHs calculated form the response to the element (***D***) and sequence (black PSTH; ***E***) situation. The time course of the median suppression is plotted in gray. Note that strong suppression occurs during and directly after high activity rates. The latter suppression reduces the postactivity to zero (black arrows). ***F***, Boxplots showing the increase in the signal-to-noise ratio in the sequence situation compared to the element situation. Wilcoxon signed rank test: *p* < 10^−5^. norm, normalized; SNR, signal-to-noise ratio.

Population activity maps from the IC illustrate the effect of collicular suppression on the neuronal response to the echolocation sequence (Fig. 4*A*, lower panel, *B*). In the heatmaps, each row represents a normalized PSTH from one collicular unit. Each acoustic event is reliably represented in the response patterns obtained from the element (Fig. 4*A*) and sequence situation (Fig. 4*B*). However, the neuronal response to the sequence was weaker, than the response to the element situation, as indicated by the lighter activity pattern in the heatmaps. The time course of suppression was visualized by subtracting the population activity map of the element situation from that obtained in the sequence situation (Fig. 4*B* – Fig. 4*A*= Fig. 4*C*). High suppression rates are indicated by bright spots (negative values) in Figure 4*C*. Suppression occurred mainly during and briefly after neuronal excitation, as postexcitatory suppression. The latter can be seen when comparing the median PSTHs in response to the sequence (Fig. 4*E*, black traces) and element situations (Fig. 4*D*). The suppression resulted in decreased neuronal activity peaks (compare maximum amplitudes at Fig. 4*D*,*E*). The postexcitatory suppression lowered the median spike rate to zero in some cases (Fig. 4*D*,*E*, arrow). In response to the element situation, the spiking activity rarely dropped to zero. The postexcitatory suppression increased the signal-to-noise ratio in the sequence compared to the element situation (Fig. 4*F*; median signal-to-noise ratios: 0.33 and 0.17 for sequence and element situation, respectively; Wilcoxon signed rank test: *p* < 10^−5^).

### Collicular suppression sharpens tuning to specific call-echo elements without changing tuning preference

**Figure 5.**
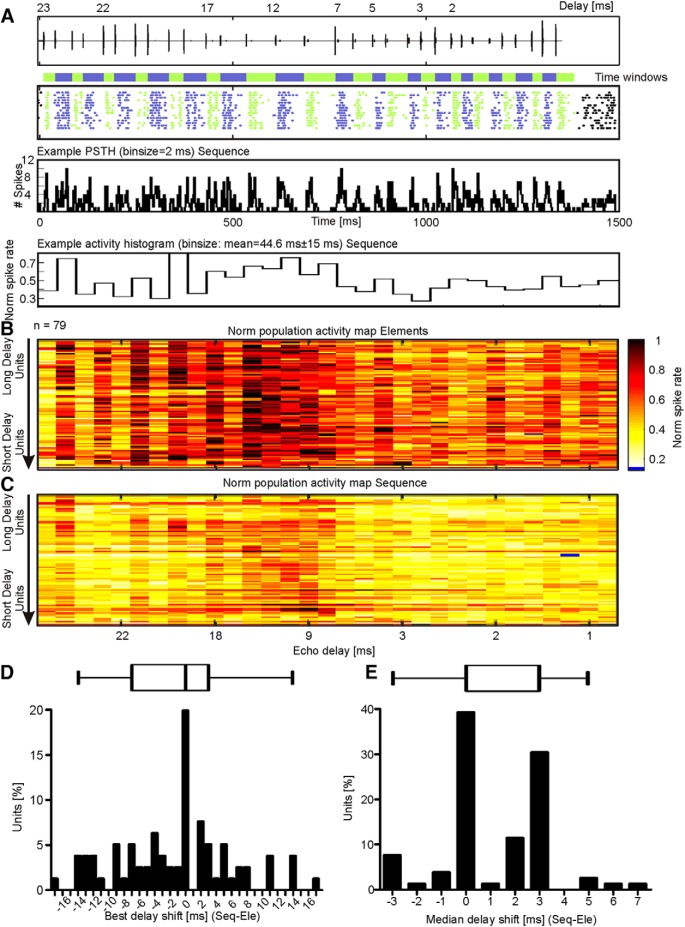
Collicular suppression sharpens neuronal tuning to call-echo elements. ***A***, Oscillogram of the echolocation sequence. Neuronal response of the example unit from Figure 3*A* is shown as raster plot and PSTH (binsize = 2 ms). To investigate neuronal tuning to certain call-echo elements, each spike was assigned according to its time point to a call-echo element. Time windows used for spike assignments to corresponding call-echo elements are indicated by alternatingly colored horizontal bars. The spikes assigned to a time window and thus to a call-echo element are correspondingly color coded. The activity rate was plotted against the call-echo elements which can be characterized based on their echo delay (*x*-axis). Note that, the depicted unit responded more strongly to long than to short delays, having its maximum response at element #8 (best delay of 20 ms). ***B***, ***C***, Normalized population activity maps in response to the element (***B***) and sequence (***C***) situation. Units were ordered along the *y*-axis according to their best delay calculated from the response to the sequence. ***D***, ***E***, Boxplots and histograms represent the best delay (***D***) and median delay shifts (***E***), calculated by subtracting the best or median delay in response to the element situation from the best or median delay in the sequence situation, respectively.

Next, we tested the influence of collicular suppression on the neuronal tuning to call-echo elements of the echolocation sequence. To assess the neuronal tuning to the call-echo elements, each spike recorded in the sequence situation was assigned to the call-echo element that putatively evoked the spike (procedure shown for one example unit in Fig. 5*A*). Each call-echo element was associated to a time window that lasted from call onset to the following call (colored, horizontal bars in Fig. 5*A* represent endpoints of each call-echo element). By using the time windows and the spike time points, each spike was assigned to a call-echo element. For instance, spikes occurring during the first time window (Fig. 5*A*, initial green spikes) were elicited by the first call-echo element. The subsequent blue spikes were putatively evoked by the second call-echo element, and so on. For visualization, alternating green and blue spikes indicate the corresponding call-echo elements to which the spikes were assigned to. Based on the spike assignment, the PSTHs could be transformed into activity histograms (Fig. 5*A*, low panels). The activity histograms of all collicular units were represented as a color-coded population activity map (Fig. 5*B*,*C*). The calculated call-echo element activity maps showed a clear selectivity toward certain call-echo elements. Strong neuronal activity was elicited by the call-echo elements representing intermediate to long echo delays (9-20 ms; Fig. 5*B*,*C*). Note that the units were sorted in descending order according to the call-echo element eliciting the highest spike rate in response to the sequence situation. Long-delay tuned neurons were positioned at the top and short-delay tuned neurons at the bottom of the activity maps in Figure 5*B*,*C*. In the sequence situation, collicular suppression lowered the neuronal activity (Fig. 5*C*, brighter, more yellowish color). Suppression also sharpened the neuronal tuning to certain call-echo elements. The sharpening did not significantly change the best delays of the collicular neurons when considering all recorded collicular units (Fig. 5*D*; Wilcoxon signed rank test: *p* = 0.13). In contrast, median delays shifted significantly toward longer delays, indicating that the response to long delays was less suppressed than the response to short delays. In other words, the strength of suppression increased over time during the stimulus presentation, in some neurons (Fig. 5*E*; Wilcoxon signed rank test: *p* < 10 ^5^). Note that the median delay did not change in 39% of the units indicating that collicular suppression did not change delay tuning in all collicular units.

### In the IC, information from multiple objects can be processed in parallel

Recent studies showed that cortical neurons process object information from one object (usually the nearest object) when the animals are stimulated with echolocation sequences containing echo information from multiple objects (Beetz et al., 2016b; Greiter and Firzlaff, 2017). Neuronal responses to distant objects are usually suppressed. Since the present study shows that collicular suppression is weaker than cortical suppression, we were interested in determining whether echo information from multiple objects can be processed at the IC level. To address this question, the bats were presented with an echolocation sequence that contained echo information from three objects (multiple-object sequence; Fig. 1*F–I*). The neuronal response was then compared with the response evoked by stimulation with echolocation sequences containing echo information from one object only (single-object sequence). Note that single-object sequences were obtained by manually deleting object-specific echoes from the multiple-object sequence (see Materials and Methods). Thus, single-object sequences contain the same spectro-temporal information as the multiple-object sequence, except for the missing echo information from two out of the three objects. Seventy-seven collicular spike-sorted single units, with CFs higher than 35 kHz, were tested with the multiple-object sequence and each of the three single-object sequences.

**Figure 6.**
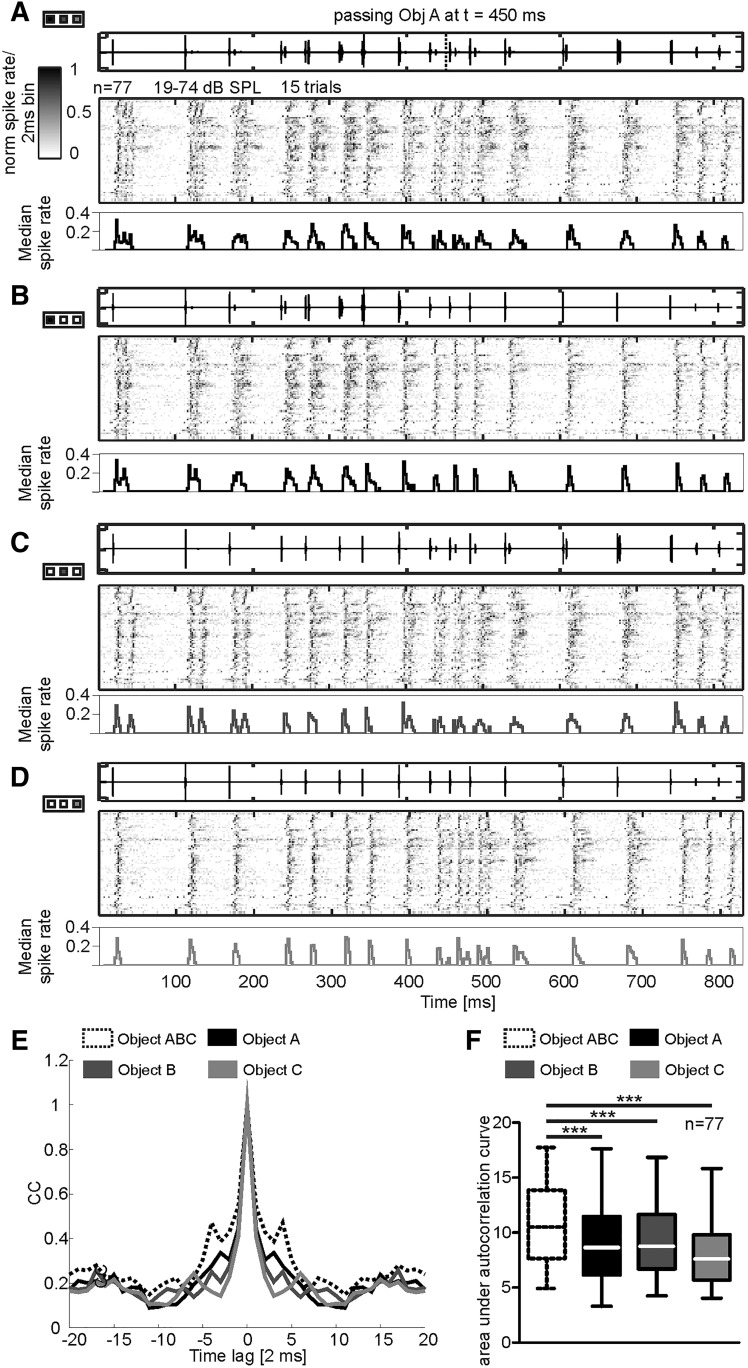
Neuronal response to the multiple-object sequence. ***A–D***, Stimulus oscillograms, population activity maps (binsize = 2 ms), and median PSTHs from 77 collicular units in response to the multiple-object (***A***), object A (***B***), object B (***C***), and object C (***D***) sequence. Legends on the left side from each oscillogram define the position of the object along the swing trajectory. Note that each acoustic event, including calls and echoes, is represented in the response pattern. ***E***, Autocorrelation functions of the PSTHs from an example unit indicated by arrows in ***A–D***. The autocorrelation function of the PSTH in response to the multiobject sequence is wider than the one of the PSTHs in response to the single-object sequences. ***E***, Statistical comparison of the area under the autocorrelation curves of 77 collicular units indicate that the response to the multiple-object sequence was broader than the response to the single-object sequences (Friedman one-way ANOVA and Dunn’s multiple comparison test: *p* < 10^−5^).

Population activity maps and the median PSTH (binsize = 2 ms) show that each acoustic event evoked a neuronal response when the bats were stimulated with the multiple-object sequence (Fig. 6*A*) and with each single-object sequence (Fig. 6*B*–*D*). The temporal bandwidth of PSTHs, obtained in response to the multiple-object sequence, was wider than the one obtained with single-object sequences. The latter becomes obvious when comparing the width of the activity peaks in the median PSTHs with each other (Fig. 6*A–D*, PSTHs in the bottom subpanels). The width of the activity peaks or the response duration was calculated by autocorrelations of the PSTHs (shown for one example unit in Fig. 6*E*). Autocorrelations were restricted to time lags of ±20 ms. The larger the area under the autocorrelation curve, the longer is the response duration. The presence of three echoes, instead of one echo, following each call increases the response duration, indicating that the collicular unit encoded echo information from more than one object. At the population level, the response duration was also significantly longer when the bats were stimulated with the multiobject sequence than with the single-object sequences (Friedman one-way ANOVA and Dunn’s multiple comparison test: *p* < 10^−5^; Fig. 6*F*).

**Figure 7.**
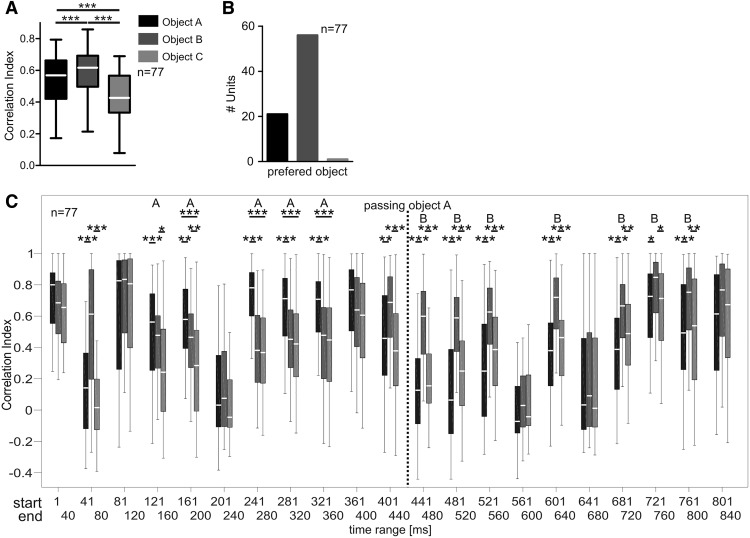
Influence of each object on the neuronal response to the multiple-object sequence. ***A***, Correlation values between each single-object PSTH (object A, object B, and object C PSTH) and the multiple-object PSTH are plotted as boxplots. Object B PSTH had the highest similarity to the multiple-object PSTH (Friedman one-way ANOVA and Dunn’s multiple comparison test: *p* < 10^−5^). ***B***, Histogram quantifying the object preference of the population of units, according to the unit’s maximum correlation index. The single-object PSTH that resembles mostly the multiple-object PSTH results into the highest correlation index. Most units showed the highest correlation index when comparing the object B with the multiple-object PSTH. ***C***, Time course of correlation indices calculated from 40-ms time windows of the PSTHs of each single-object PSTH correlated to the corresponding time window in the multiple-object PSTH. Before passing object A, the multiple-object PSTH mostly resembled the object A PSTH. Thus, object A had the highest impact on the response pattern to the multiple-object sequence. After passing object A, the multiple-object PSTH was mostly affected by object B. The letters “A” and “B” above the boxplots indicate the time windows where object A and object B led to higher correlation values, respectively. The letters A and B are temporally confined before and after passing object A, respectively. Kruskal-Wallis and Dunn’s multiple comparison *post hoc* test; *p < 0.05; **p < 0.01; ***p < 0.001.

A correlation between the PSTHs calculated in response to each single-object and the multiple-object sequence allowed for a quantification of the influence from each object on the neuronal response. Correlation values were highest between PSTHs obtained in response to object B (object B PSTHs) and PSTHs corresponding to the multiple-object sequence (multiple-object PSTH; median correlation index: 0.57 object A, 0.62 object B, and 0.42 object C; Friedman one-way ANOVA and Dunn’s multiple comparison test: *p* < 10^−5^; Fig. 7*A*). Thus, the neuronal response to the multiple-object sequence mostly resembles the response to the object B sequence. This result is not surprising because object B contributes more echoes to the multiple-object sequence (17 echoes) than object A (eight echoes) or object C (seven echoes). Thus, the highest stimulus similarity was already biased toward object B in the multiple-object sequence. Note that stimulus similarity does not exclusively account for the differences in the calculated correlation values (Fig. 7*A*). Object A and object C provide about the same number of acoustic events to the multiple-object sequence. If the correlation values were simply reflecting the amount of acoustic events, then object A and object C should comparably influence the response to the multiple-object sequence. However, object A PSTHs were more similar to the multiple-object PSTH than object C PSTHs (Fig. 7*A*). Note that the intensity of the echoes from object A were usually higher than from object C. Therefore, intensity driven influences cannot be excluded with the stimulus setting used in the present study. Next, we determined which of the three objects influenced most the multiobject-evoked PSTH. The object resulting in the highest correlation value was determined for each unit. As expected from the previous results, object B had the highest impact in the response to the multiple-object sequence in most neurons (73%; Fig. 7*B*). However, the multiobject PSTH was most similar to the object A and object C PSTHs in 27% and 1% of the units, respectively.

In the next step, we tested whether the collicular neurons shifted their tuning preferences to the objects, during the presentation of the multiple-object sequence. For quantification, the PSTHs (binsize = 2 ms) were cut into 21 PSTH segments. Each PSTH segment contained 40 ms of neuronal activity. Note that the 40-ms time window correspond to the time window used for the autocorrelation analysis in Figure 6. Correlation values between the segmented multiple-object PSTH and each segmented single-object PSTH were plotted as boxplots as a function of temporal position of each segment in the sequence (Fig. 7*C*). Before passing object A at the 450-ms mark (Fig. 7*C*, black vertical dashed line), object A most strongly determined the neuronal response to the multiple-object sequence. This is indicated by significantly higher correlation values in five out of eleven PSTH segments (Fig. 7*C*, depicted by an “A” above the boxplots). After passing object A, only echoes from object B and object C were present. In that situation, object B had the strongest influence on the multiple-object PSTH, as indicated by higher correlation values in seven out of ten PSTH segments (Fig. 7*C*, depicted by a “B” above the boxplots). Correlation values between the segmented object C PSTH and the segmented multiple-object PSTH were never significantly higher than the ones between the segmented multiple-object PSTH and the segmented object A and object B PSTHs. In summary, the initial 450 ms of the response pattern to the multiple-object sequence is predominantly determined by echo information coming from object A. After passing object A, echoes from object B have the strongest influence on the response pattern to the multiple-object sequence. Although collicular neurons responded to each echo of the multiple-object sequence, collicular suppression ensures that echoes from the nearest object are mostly determining the response pattern to the multiobject sequence.

## Discussion

In the auditory system, neuronal spikes are usually synchronized to the stimulus envelope. Along the ascending auditory pathway, the cutoff frequency that can elicit such synchronization decreases (for review see: (Joris et al., 2004; Wang et al., 2008; Simmons and Simmons, 2011). When stimulating animals with acoustic rates higher than 40 Hz, cortical neurons are sometimes completely suppressed. Thus, neuronal suppression degrades temporal processing of repetitive stimuli. With this in mind, one could ask how neurons of animals that behaviorally rely on high repetition rates of sensory information cope with such suppression effect? The present study quantified the influence of the temporal context (present in natural echolocation sequences) on the response of IC neurons of bats. The echolocation sequences used by us mimic a stimulus situation encountered by the bat when approaching one or several target objects.

### Neuronal suppression does not necessarily degrade temporal processing

The cutoff frequencies of mammalian collicular neurons are heterospecific and range between 10 and 1000 Hz [∼100–150 Hz gerbils (Krishna and Semple, 2000), and guinea pigs (Rees and Palmer, 1989), squirrel monkeys (Müller-Preuss et al., 1994), ∼200 Hz in rats (Rees and Møller, 1983), and ∼1000 Hz in cats (Langner and Schreiner, 1988)]. Cutoff frequencies of bat collicular neurons range between 94 and 400 Hz [∼100 Hz in *Rinolophus rouxi* (Reimer, 1987) and ∼94–400 Hz in *M. lucifigus* (Condon et al., 1994)]. Overall, the results from previous studies would suggest that bat IC neurons should be able to synchronize their spiking to echolocation sequences in which the repetition rate never reaches 100 Hz, as it is the case in *C. perspicillata*. Our results confirm this prediction. We show that collicular neurons of *C. perspicillata* synchronize their discharges to the stimulus envelope of each acoustic signal in the echolocation sequences (Fig. 3). Note that this result is contrasted by findings from studies in the Mexican free-tailed bat, which show that some collicular neurons respond selectively to particular syllables of a communication sequence (Andoni and Pollak, 2011). This discrepancy could arise from heterospecific effects or from using different types of acoustic stimuli, i.e., echolocation compared to communication signals.

Despite the neuronal synchronization in the IC of *C. perspicillata*, the collicular neurons were suppressed in the sequence situation. However, instead of degrading temporal processing, collicular suppression improved temporal processing by increasing the signal-to-noise ratio (Fig. 4) and the neuronal selectivity to particular call-echo elements (Fig. 5). In agreement with our results, numerous studies have reported that subcortical neurons sharpen their neuronal tuning with increasing repetition rates [IC: *Myotis lucifugus* (Friend et al., 1966; Galazyuk et al., 2000), *E. fuscus* (Pinheiro et al., 1991; Chen and Jen, 1994; Moriyama et al., 1994; Wu and Jen, 1996; Jen and Chen, 1998; Jen and Zhou, 1999; Jen et al., 2001; Zhou and Jen, 2001; Zhou and Jen, 2002; Sanderson and Simmons, 2005; Wu and Jen, 2006; Zhou and Jen, 2006; Jen and Wu, 2008); superior colliculus: *E. fuscus* (Valentine and Moss, 1997; Wohlgemuth and Moss, 2016)]. Some studies even described repetition rate selective neurons in the IC of insectivorous bats, like *E. fuscus*, (Pinheiro et al., 1991; Sanderson and Simmons, 2005) and *M. lucifugus* (Condon et al., 1994). Repetition rate or inter-syllable interval selective neurons have been described in different animals, including crickets (Zorovic and Hedwig, 2011), fish (Crawford, 1997), frogs (Rose, 2014; Rose et al., 2015), and birds (Araki et al., 2016). Crickets (Hedwig, 2006), frogs (Feng et al., 1990), and presumably birds (Araki et al., 2016) identify conspecifics by determining species-specific repetition rate or inter-syllable interval of the acoustic signals. In bats, an improvement in temporal tracking with signal repetition rate could be of advantage for information extraction during echolocation. Some bat species increase their call rate from 10-200 Hz during an approach flight that ends with an insect capture (Simmons et al., 1979). Thus, specific neuronal populations are excited at different hunting stages (Jen and Schlegel, 1982; Condon et al., 1994). In contrast to insectivorous bats, frugivorous bats such as *C. perspicillata* change less dramatically their call rates during echolocation [*C. perspicillata* (Thies et al., 1998), *Phyllostomus discolor* (Linnenschmidt and Wiegrebe, 2016)]. During approach flights, *C. perspicillata* increases the call rate from 12 ± 19 to 24 ± 39 Hz (Thies et al., 1998). Although frugivorous bats change their call rates less prominently than insectivorous bats, the former could still profit from and enhanced temporal representation of acoustic streams at the level of the IC.

### Temporal selectivity increases from the IC to the AC

By using the same stimulus settings in the IC and AC (Beetz et al., 2016a), it is possible to compare the influence of a natural temporal context in both brain areas for the first time. Cortical neurons of *C. perspicillata* have a cutoff frequency of 20 Hz (Martin et al., 2017). To modulation (Martin et al., 2017) or repetition rates (Beetz et al., 2016a; Hechavarría et al., 2016b) higher than 20 Hz, cortical neurons become suppressed and they respond exclusively to certain call-echo elements (Beetz et al., 2016a). The results from this study show that collicular suppression is weaker than the cortical one (Fig. 3*C*) and that main suppressive effects seen in the AC may arise at the thalamus or cortex, as proposed by findings from rodents (Wehr and Zador, 2005; Bayazitov et al., 2013). Note that although suppression is weaker in the IC, the time course of suppression is comparable between the cortex and midbrain (for IC data, see gray trace in Fig. 4*E*). Main suppressive effects occurred during or directly after strong excitations. Postexcitatory suppression is common in the mammalian cortex (Suga et al., 1983; Joris et al., 2004; Beetz et al., 2016a) and has also been described in the IC of different bat species, including *E. fuscus* (Covey et al., 1996), *Tadarida brasiliensis mexicana* (Bauer et al., 2000), and *M. lucifugus* (Voytenko and Galazyuk, 2007).

An increase of neuronal selectivity for temporal parameters or specific vocalizations along the processing pathway has been demonstrated in different animals, including crickets (Schildberger, 1984; Zorovic and Hedwig, 2011; Kostarakos and Hedwig, 2012; Schöneich et al., 2015), fish (Partridge et al., 1981), frogs (Rose and Capranica, 1983; Feng et al., 1990), birds (Margoliash, 1986; Doupe and Konishi, 1991; Lewicki and Arthur, 1996; Fujimoto et al., 2011; Theunissen and Elie, 2014), and mammals (Wang et al., 1995; Beetz et al., 2016a). Findings from the AC of rats demonstrated that hearing selectivity is refined by selective inhibition during development (Chang et al., 2005). The present results from *C. perspicillata* indicate that hearing selectivity along the ascending auditory pathway could be a least partially shaped by time-dependent neuronal suppression. We base this claim on the fact that neuronal selectivity to particular call-echo elements was higher in the sequence than in the element situation (present study and Beetz et al., 2016a). The latter holds true for both the IC and AC, but suppression does have the strongest effects at the level of the AC.

### Relevance of the stimulus history for neuronal processing

The sensory world continuously changes and preceding stimuli determine how subsequent stimuli are processed (Brosch et al., 1999; Näätänen et al., 2001; Bartlett and Wang, 2005; Dehaene et al., 2015). In birds, the significance of stimulus history, represented by the temporal context and stimulus order, has been widely demonstrated in neurophysiological experiments. Neurons of the auditory forebrain, respond most strongly to syllables of the bird’s own song when presented in the natural temporal context (Margoliash, 1983, 1986; Margoliash and Fortune, 1992). The neurons respond less selectively when presenting the syllables temporally isolated (Margoliash, 1983; Margoliash and Fortune, 1992), as in the element situation of the present study, or when presenting the bird’s song in a reversed manner (Margoliash, 1986; Margoliash and Fortune, 1992; Volman, 1996). In mice, neurophysiological experiments demonstrated that cortical neurons respond most strongly to ultrasonic vocalizations when the mice were stimulated with a natural temporal context (Carruthers et al., 2013). In bats, the neuronal selectivity to echolocation (present study and Beetz et al., 2016a) and communication signals (Esser et al., 1997; Hechavarría et al., 2016b) is also highest when stimulating with the natural temporal context.

Although, presenting the call-echo elements in a chronological order, in the present study, it is possible that neuronal tuning could additionally depend on the order of call-echo elements in the sequence. Order selective neurons have been characterized in rats (Kilgard and Merzenich, 2002; Nakahara et al., 2004), birds (Lewicki and Arthur, 1996; Doupe, 1997), and monkeys (Ninokura et al., 2004; Yin et al., 2008; Sadagopan and Wang, 2009; Berdyyeva and Olson, 2010; Crowe et al., 2014). The present study demonstrates the importance of not only using natural stimuli but also presenting the stimuli in the natural temporal context (Theunissen and Elie, 2014).

### Target distance processing in the IC of *C. perspicillata*

To our knowledge, this is the first study characterizing the properties of delay tuning in the IC of *C. perspicillata*. In *Pteronotus parnellii*, delay tuned neurons of the IC usually respond only to a combination of call and echo but not, or only sparsely, to call or echo alone (Yan and Suga, 1996; Macías et al., 2016). In contrast, collicular neurons of *E. fuscus*, another insectivorous species respond in 59% of the recordings to call and echo (Sanderson and Simmons, 2005). In *C. perspicillata*, the collicular units usually respond to call and echo which is indicated by two activity peaks per call-echo element in the median PSTHs (Fig. 4*D*,*E*). Neuronal tuning to certain call-echo elements was only visible when integrating the number of spikes elicited by each call-echo element.

In the midbrain of *E. fuscus* best delays shorter than 8 ms are rare (IC: Dear and Suga, 1995; superior colliculus: Valentine and Moss, 1997). We also did not find best delays shorter than 8 ms in the present study (mean “best delay” = 14.4 ± 6 ms; mean “median delay” = 10.5 ± 2.1 ms; Fig. 5*C*). However, delay tuning to short delays has been well characterized in the mustached bat’s IC (Mittmann and Wenstrup, 1995; Portfors and Wenstrup, 1999; Wenstrup and Portfors, 2011). These different findings may be due to interspecific differences. Although, it is tempting to compare the present results with studies on delay tuning from other bat species, it is noteworthy that we characterized delay tuning based on natural acoustic stimuli while previous studies used mostly artificial signals that mimicked the bat’s call-echo pairs presented in isolation (Kössl et al., 2014). Contrary to the IC, in the cortex of *C. perspicillata*, a number of neurons do respond to echo delays shorter than 8 ms. The latter occurs regardless of whether natural sequences or artificial pulse-echo elements are used for calculating the tuning (Hagemann et al., 2011; Beetz et al., 2016a). Future studies could assess whether the short-delay tuning found in *C. perspicillata*’s AC is created along the colliculo-cortical axes, or whether it exists in regions of the IC that were not targeted in the present study. Previous studies have shown that in the cortex, GABA-mediated inhibition can change the best delay of the neurons (Xiao and Suga, 2004; Hechavarría and Kössl, 2014). The latter could be a mechanism for modifying the delay tuning that is already established at the level of the IC.

### Collicular responses to multiple echoes

Only a few studies have characterized how bat auditory neurons extract information from multiecho biosonar sequences (Edamatsu and Suga, 1993; Sanderson and Simmons, 2002; Beetz et al., 2016b; Greiter and Firzlaff, 2017). This stimulus setting mimics the presence of multiple objects or multi-reflective surfaces. One study in the IC of *E. fuscus* tested the neuronal processing of “spectral notches.” Such spectral notches derive from two temporally overlapping echoes (Sanderson and Simmons, 2000). Note that in the preset study spectral notches do not occur since temporally nonoverlapping echoes were used to create our stimulation sequence. Nonoverlapping echoes were created by the presence of up to three objects located at different distances from each other.

Previous data from the AC of *C. perspicillata* showed that the neurons preferentially process echo information from the nearest object (Beetz et al., 2016b). The data from anaesthetized bats imply that neurons focus by default on the nearest object and that this processing strategy works without the attention of the animal. However, recent behavioral experiments from *Pipistrellus kuhlii* (Amichai and Yovel, 2017) and from *P. abramus* (Fujioka et al., 2016) demonstrate that bats can attend to distant objects, even in the presence of immediate objects. Such behavioral results clearly go beyond the neurophysiological results from the anaesthetized bat that was stimulated with a natural echolocation sequence (Beetz et al., 2016b). Although the animal’s attention could affect neuronal processing, the behavior observed in *P. kuhlii* and *P. abramus* could also be accomplished with neuronal information from the IC. Collicular neurons keep track of echoes from multiple objects (Fig. 6). In the AC of *P. discolor*, most neurons also respond to the nearest object, but a small population of neurons preferably responded to a more distant object (Greiter and Firzlaff, 2017). These results resemble more the IC results of the present study than the AC results from *C. perspicillata* (Beetz et al., 2016b). The different results from *C. perspicillata* and *P. discolor* could be based on heterospecific differences but differences in the used acoustic stimuli cannot be discarded. Natural hearing is an active process that requires the animal’s attention (Theunissen and Elie, 2014) and neurons involved in auditory feedback in self-vocalizations have been characterized (Schuller, 1979; Radtke-Schuller and Schuller, 1995). Therefore, behavioral results should be cautiously correlated with neurophysiological results from anaesthetized and passively listening bats.

### Neuroethological roles of the IC for echolocation in bats

Bilateral ablation experiments of the main nucleus of the IC showed that the IC is required for echolocation (Suga, 1969b). In comparison, cortical ablation and focal inactivation of the AC less severely affected the bats’ echolocation behavior (Suga, 1969a; Riquimaroux et al., 1991). The IC projects to and receives input from different motor centers (Schweizer, 1981; Covey et al., 1987; Olazábal and Moore, 1989; Moriizumi and Hattori, 1991; Schuller et al., 1991; Wenstrup et al., 1994). Therefore, the IC is discussed to be important for control of fast motor commands and reflexive behaviors during echolocation (Casseday and Covey, 1996). The results presented in this study corroborate this idea because the temporal structure of the echolocation sequence is highly preserved at the collicular level. During echolocation, bats need to integrate and possibly predict echo information. Collicular neurons convey time stamps of the echolocation signals. The latter could be important for predictive coding in high brain areas (Wacongne et al., 2012). Despite the high tracking ability of collicular neurons (Figs. 3, 4), IC units were selective to specific call-echo elements (Fig. 5) and to object-specific echoes (Fig. 7). Based on our findings, one could speculate that IC responses allow parallel processing of multiple auditory streams, with a certain selectivity to specific echo delays.

## Synthesis

Reviewing Editor: Darcy Kelley, Columbia University Decisions are customarily a result of the Reviewing Editor and the peer reviewers coming together and discussing their recommendations until a consensus is reached. When revisions are invited, a fact-based synthesis statement explaining their decision and outlining what is needed to prepare a revision will be listed below. The following reviewer(s) agreed to reveal their identity: Andrea Simmons, Khaleel Razak.

The authors examine the coding of naturalistic echolocation sequences in the inferior colliculus (IC) of anesthetized Carollia, and compare these responses with those recorded earlier from the same species in the auditory cortex (AC). Methods are clear and the data have been analyzed appropriately (however see below). The figures are clear and informative.

This study examined responses of neurons in the central nucleus of the inferior colliculus (IC) to address how response magnitudes are modulated by a natural echolocation sequence compared to the same sounds presented randomly and at slower repetition rates. These recordings were obtained from C. perspicillata bats that were anesthetized and compared to data obtained using a similar paradigm from the auditory cortex. The main findings reported are that the natural sequence elicits more suppression compared to segmented presentation, resulting in better signal to noise ratio in the responses. In addition, the suppression and the difference between stimulus paradigms in the IC were stronger than that in the cortex. One other major finding is that, in the presence of echoes from multiple objects, the IC neurons provide better tracking than cortical neurons. The data are well presented for visualization and the quantification is appropriate.

From a broad auditory neuroscience perspective, these data are not surprising given the broad array of studies that have shown across multiple species that repetition rate influences response magnitudes and that the IC can track faster rates than the cortex. Almost all of the data in this paper can be explained based on that general understanding. The authors provide a neuroethological framework for this phenomenon suggesting that tracking of fast repetition rates, to the extent that it drives behaviors in bats, can be achieved based on IC neuron output. However, cortical population level responses can make similar contributions in terms of tracking fast repetition rates. Overall, the study adds another species to the general hierarchical repetition rate changes known to be consistent in auditory system.

The study addresses an interesting neuroethological question, and replicates previous findings.

A shared concern of the reviewers is that, though the data are new in terms of IC recordings from this particular species of bat, the ideas motivating the work are not novel. The authors do not appropriately acknowledge published work in other species of bat that is of obvious intellectual and methodological relevance. The introduction should be rewritten to include the appropriate citations, and to make it clear how the present work fits in with this earlier literature.

Another major comment is that echolocation is an active process in which the bat's nervous system has some information regarding when a call is made. The recordings were all done in anesthetized bats, which remove some of the active component aspects of echolocation signal processing. The authors need to comment on this discrepancy between purported function in echolocation and the neural responses.

Stylistic comment: Please ensure that the use of commas is consistent with American English. The manuscript can be improved significantly with an edit for grammar, removal of vague statements and better explanation of methods

.

Specific comments

Significance

Line 22: “that can be described” instead of “that can be circumscribed“

Line 27: “allows for a high”

Introduction

Line 31-32: suppress where?

Line 40: substitute “selectively” for “in a facilitatory (misspelled in manuscript) manner”

Line 54 - expected from what? More explanation...

Line 56 - period

Line 62-63 - this is a well established fact across multiple species - need citations. The sequence vs. element difference is likely carried by repetition rate differences.

Methods

Line 101-102: grammar

Line 197: precisely?

Line 200-202 - separate paragraph not needed.

Line 201 - rationale for using different bin sizes?

Line 208-209 - this is unclear. More explanation is needed.

Results

Line 245 - how were 'w' vs. 'v' shaped tuning curves quantified? The transition can be continuous between these shapes.

Line 256: grammar - also 'stimulus history' may be overstating this. Repetition rate can explain. While repetition rate can be thought of as stimulus history, I believe the phrase indicates something more.

Figure 5 - legends - 'barely' in the title is vague.

Line 802-805 - red vs green? Also this analysis is unclear.

Line 322-325 - instead of % of neurons shifting, a paired t-test using each neuron's sequence vs. element response may be more appropriate to indicate whether 'delay tuning' changes significantly. The present description is vague. For e.g., what constitutes a 'shift'?

Line 355 - sentence end is abrupt

Line 367 - period

Line 392 - 'more strongly processed' - what does this mean? How was it quantified? How can you interpret this way based on what was quantified?

Discussion

Overall the discussion is highly 'bat-centric' and needs to argue why these findings could be of general interest.

Line 415-416 - Immunostaining is not going to provide the answer to the question posed.

Line 423-425 - Does 2005 count as recent?

Line 425-427 - 'not surprising' - context is poorly established. The 2 bat species described in previous sentences are different. The present bat species is like one of them. So surprising or not depends on the comparison species - which should be done based on phylogeny.

Line 429-430 - grammar

Line 453 and 469- extra ')'

Line 517 - 'of' instead of 'on'

References

Simmons & Simmons 2011a and 2011b is the same paper; Kossl et al. 2014a and 2014b is the same paper.

## Author Response

Institut für Zellbiologie und Neurowissenschaft

Max-von-Laue-Straße 13

60438, Frankfurt/Main, Germany

Tel.: +49 69798 42066

Dear, Prof. Dr. Kelley,

we are thankful for your input and for the reviewers' valuable comments on the manuscript and we appreciate to have received the chance to revise our manuscript for resubmission.

As suggested, changings in the revised manuscript are indicated in “red”.

Below you will find a point-by-point reply to your and the reviewers questions and suggestions.

Please, note that the introduction and discussion of the manuscript were almost completely rewritten. Thus, some specific comments on the introduction and discussion did not apply.

Please, feel free to contact me in the case of any upcoming questions about the revised manuscript.

Best regards,

M. Jerome Beetz

Mailing address:

M. Jerome Beetz

Email: beetzjerome@gmail.com

Institut für Zellbiologie und Neurowissenschaft, Max-von-Laue-Straße 13, 60438, Frankfurt/Main, Germany, Tel.: +49 69798 42066

-

Synthesis of Reviews:

Significance Statement Comments for Author (Required):

Significance is clear,

Comments on the Visual Abstract for Author (Required):

Visual abstract is informative.

Synthesis Statement for Author (Required):

The authors examine the coding of naturalistic echolocation sequences in the inferior colliculus (IC) of anesthetized Carollia, and compare these responses with those recorded earlier from the same species in the auditory cortex (AC). Methods are clear and the data have been analyzed appropriately (however see below). The figures are clear and informative.

This study examined responses of neurons in the central nucleus of the inferior colliculus (IC) to address how response magnitudes are modulated by a natural echolocation sequence compared to the same sounds presented randomly and at slower repetition rates. These recordings were obtained from C. perspicillata bats that were anesthetized and compared to data obtained using a similar paradigm from the auditory cortex. The main findings reported are that the natural sequence elicits more suppression compared to segmented presentation, resulting in better signal to noise ratio in the responses. In addition, the suppression and the difference between stimulus paradigms in the IC were stronger than that in the cortex. One other major finding is that, in the presence of echoes from multiple objects, the IC neurons provide better tracking than cortical neurons. The data are well presented for visualization and the quantification is appropriate.

From a broad auditory neuroscience perspective, these data are not surprising given the broad array of studies that have shown across multiple species that repetition rate influences response magnitudes and that the IC can track faster rates than the cortex. Almost all of the data in this paper can be explained based on that general understanding. The authors provide a neuroethological framework for this phenomenon suggesting that tracking of fast repetition rates, to the extent that it drives behaviors in bats, can be achieved based on IC neuron output. However, cortical population level responses can make similar contributions in terms of tracking fast repetition rates. Overall, the study adds another species to the general hierarchical repetition rate changes known to be consistent in auditory system.

The study addresses an interesting neuroethological question, and replicates previous findings.

A shared concern of the reviewers is that, though the data are new in terms of IC recordings from this particular species of bat, the ideas motivating the work are not novel. The authors do not appropriately acknowledge published work in other species of bat that is of obvious intellectual and methodological relevance. The introduction should be rewritten to include the appropriate citations, and to make it clear how the present work fits in with this earlier literature.

Response: We rewrote the introduction of the manuscript to present our research question into a broader biological perspective.

In the following, we summarize the novelty of our results.

Our main goal was to investigate how natural echolocation sequences are processed in the IC and which influence the natural temporal context has on the neuronal tuning. Previous studies tested the influence of the stimulus rate on the neuronal tuning in the IC and AC of different bat species. In contrast to our stimulus setting, most previous studies that tested the influence of the stimulus rate on the neuronal tuning varied the stimulus rate in a consistent manner. In other words, they tested one stimulus rate after the other. However, the stimulus rates, or call-repetition rates, vary during echolocation, especially with respect to an approach flight. The call-rate is usually increased when the bat is approaching a target. Variably changing call-rates have only been tested in the superior colliculus and IC of the insectivorous bat Eptesicus fuscus (Sanderson and Simmons, 2005; Wohlgemuth and Moss, 2016) and in the AC of the frugivorous bats Carollia perspicillata (Beetz et al., 2016) and Phylostomus discolor (Bartenstein et al., 2014). So far, no study tested how natural echolocation sequences are processed in the IC and compared the data with corresponding data from the AC. To our knowledge, the comparison between subcortical (IC) and cortical (AC) responses to natural stimuli in bats has not been conducted. This is an important comparison, as it could shed light into the transformation that occur along the ascending auditory pathway in terms of sensory information coding.

In contrast to the bat literature, in frogs (Feng et al., 1990) and birds (Theunissen and Elie, 2014), a comparison between the neuronal response to natural acoustic stimuli from the IC (or MLd in birds) to the AC (auditory forebrain like HVc, auditory area field L in birds) has been reported in the last years. Corresponding to our results, they showed that collicular neurons are usually less specifically tuned than cortical neurons (except for some repetition rate or interval-counting neurons from frogs (Rose, 2014). The increased selectivity of temporal processing from the IC to the cortex, reported in other species, go in line with our results from the bat. We hypothesize that different strengths of suppression in the IC and AC determine the temporal filters and thus, the neuroethological function of these two brain areas.

We hope that our results can motivate researchers studying the auditory system to use not only natural stimuli but also a natural temporal context for investigating neuronal processing in different animal species.

Another major comment is that echolocation is an active process in which the bat's nervous system has some information regarding when a call is made. The recordings were all done in anesthetized bats, which remove some of the active component aspects of echolocation signal processing. The authors need to comment on this discrepancy between purported function in echolocation and the neural responses.

Response: In this version, we discuss the effect of anesthesia and passively listening bats in more detail in the discussion section. We are aware of the necessity of recording from actively behaving bats in future studies, and in this version, we emphasize the relevance of this issue.

We wrote the following in the discussion: “Natural hearing is an active process that requires the animal's attention (Theunissen and Elie, 2014) and neurons involved in auditory feedback in self-vocalizing bats have been characterized (Schuller, 1979; Radtke-Schuller and Schuller, 1995). Therefore, behavioral results should be cautiously correlated with neurophysiological results from anaesthetized and passively listening bats.” (line 547-551)

Stylistic comment: Please ensure that the use of commas is consistent with American English. The manuscript can be improved significantly with an edit for grammar, removal of vague statements and better explanation of methods

.

Response: We thoroughly checked the manuscript for grammatical issues and simplified some of the sentences. We also provide a better explanation of the methods.

Specific comments

Significance

Line 22: “that can be described” instead of “that can be circumscribed”

Response: fixed

Line 27: “allows for a high”

Response: fixed

Introduction

Line 31-32: suppress where?

Response: “Though fast acoustic repetition rates are important for many animal species, encoding these fast time-varying signals is challenged by the fact that repetitive stimuli often degrade temporal processing along the auditory pathway by evoking neuronal suppression from the auditory nerve on (Harris and Dallos, 1979; Wiggs and Martin, 1998; Joris et al., 2004; Grill-Spector et al., 2006).” (line 35-38)

Line 40: substitute “selectively” for “in a facilitatory (misspelled in manuscript) manner”

Response: We changed the sentence as suggested: “Neurons involved in distance processing respond selectively to call echo pairs, in which the echo follows the call with a certain delay (Grinnell, 1963; Feng et al., 1978; Suga et al., 1978; Suga and O'Neill, 1979; Hagemann et al., 2010).” (line 57-59)

Line 54 - expected from what? More explanation...

Response: we deleted the sentence

Line 56 - period

Response: fixed

Line 62-63 - this is a well established fact across multiple species - need citations. The sequence vs. element difference is likely carried by repetition rate differences.

Response: We added the corresponding information from the literature in the introduction and discussion.

Methods

Line 101-102: grammar

Response: Sentence changed to: “Consistent with findings in freely flying bats (Thies et al., 1998), the call duration decreases as the bat approaches the object in the pendulum paradigm (Figure 1 A).” (line 110-112)

Line 197: precisely?

Response: we deleted the sentence

Line 200-202 - separate paragraph not needed.

Response: fixed

Line 201 - rationale for using different bin sizes?

Response: Thank you very much for that comment. We just realized that the different binsizes used for the PSTHs of cortical and collicular neurons were only for visualization reasons in figure 3A and 3B. The quantitative comparison between cortical and collicular neurons (the suppression rate and the cross-correlation calculations to the stimulus envelope) were computed by using the PSTHs with a binsize of 1 ms, as it had been initially explained in the manuscript. We apologize for that mistake.

Line 208-209 - this is unclear. More explanation is needed.

Response: We rephrased the paragraph as follow: “The calculation of the signal-to-noise-ratio (Figure 4F) was based on normalized PSTHs. PSTHs were normalized in a unit-specific manner, relative to each unit's maximum spike count per bin when considering both the element and sequence situations. To distinguish between stimulus evoked responses (signal) and background activity (noise), a threshold was set to 50% of the maximum value of the normalized PSTHs. Bins crossing that threshold were defined as the “signal” and compared to the remaining bins that represent the “noise”. The signal-to-noise ratio of a call-echo element represents the sum of the number of spikes in bins defined as signals divided by the total number of spikes elicited by that call-echo element. Thus, a signal-to-noise ratio of 1 indicates that each spike elicited in response to the call-echo element, was assigned to a “signal” and that the “noise” level was zero. A signal-to-noise ratio was quantified for the responses to each call-echo element. For obtaining a unit specific signal-to-noise ratio, we calculated the median values of the signal-to-noise ratios calculated in response to each call-echo element.” (line 204-214)

Results

Line 245 - how were 'w' vs. 'v' shaped tuning curves quantified? The transition can be continuous between these shapes.

Response: The idea of a continuous transition of the frequency tuning curves is totally valid. Because the present study was not focused on the description of the receptive fields of the collicular neurons we did not quantitatively analyzed the shape of the tuning curve. Classification was just based on a qualitative analysis of the tuning curves. For us, it was just remarkable and thus necessary to point out that the frequency tuning curves of the high frequency tuned neurons of the IC had a similar shape as the ones previously described in the cortex of C. perspicillata. Instead of writing about “W”-shaped frequency tuning curves we now point out that in addition to the sensitivity to high frequencies, the high frequency tuned neurons had also a low frequency tail in their receptive fields.

Additionally, we decided to delete the discussion part describing the frequency tuning properties in the IC. To appropriately discuss the “W” and “V” tuning curves in the IC and how they are generated it is necessary to analyze the frequency receptive fields of the present study in more detail. But, as aforementioned, this was not the main scope of the present study.

Line 256: grammar - also 'stimulus history' may be overstating this. Repetition rate can explain. While repetition rate can be thought of as stimulus history, I believe the phrase indicates something more.

Response: We agree with the statement and think that the repetition rate represents only one way to test for stimulus history. For clarity, we wrote a section about the “Relevance of the stimulus history for neuronal processing” in the discussion because we want to emphasize the importance of presenting stimuli in a natural temporal context in future neurophysiological studies.

Besides, we rephrased the result section in the following way: “We analyzed neuronal responses from 79 collicular units that were recorded while the bats were listening to an echolocation sequence (“sequence situation”; black raster and PSTH (binsize = 2 ms) in figure 3A). The sequence mimicked a stimulus scenario that the bat could perceive when flying towards an object. To quantify the influence of the temporal context of the sequence on the neuronal response, the bats were also stimulated with the temporally isolated call-echo elements of the sequence. Temporal isolation means that the call-echo elements were randomly presented with a 400 ms inter-element interval (element situation; black and gray raster and gray PSTH in figure 3A).” (line 251-257)

Figure 5 - legends - 'barely' in the title is vague.

Response: We changed the title of the figure into: “Collicular suppression sharpens neuronal tuning to call-echo elements”

Line 802-805 - red vs green? Also this analysis is unclear.

Response: We changed “orange” into “blue” and made the “green” color a little brighter to get a better color contrast. We rephrased the paragraph in the result section and explained in more detail how we obtained the activity histograms. Additionally, we replaced the dashed, colored vertical lines in the oscillogram in figure 5A with colored horizontal lines that signal the extent of each response window.

The result section states the following: “To assess the neuronal tuning to the call-echo elements, each spike recorded in the sequence situation was assigned to the call-echo element that putatively evoked the spike (procedure shown for one example unit in figure 5A). Each call-echo element was associated to a time window that lasted from call onset to the following call (colored, horizontal bars in figure 5A represent endpoints of each call-echo element). By using the time windows and the spike time points, each spike was assigned to a call-echo element. For instance, spikes occurring during the first time window (initial green spikes in Figure 5A) were elicited by the first call-echo element. The subsequent blue spikes were putatively evoked by the second call-echo element, and so on. For visualization, alternating green and blue spikes indicate the corresponding call-echo elements to which the spikes were assigned to.” (line 304-312)

Line 322-325 - instead of % of neurons shifting, a paired t-test using each neuron's sequence vs. element response may be more appropriate to indicate whether 'delay tuning' changes significantly. The present description is vague. For e.g., what constitutes a 'shift'?

Response: We changed the text as suggested and made a Wilcoxon signed rank test to compare statistically delay tuning shifts induced by the sequence situation. According to the population of collicular units, we found no significant change of the best delays. But, we found significant median delay shifts towards longer delays which can be explained with a time varying suppression strength. Neurons changing their median delay towards longer delays might be more suppressed in the late phases of the sequence than at earlier phases. Thus, in some neurons the suppression strength seems to build up during the stimulus presentation.

The result section was changed as follows: “The sharpening did not significantly change the best delays of the collicular neurons when considering all recorded collicular units (Figure 5D; Wilcoxon signed rank test: p = 0.13). In contrast, median delays shifted significantly towards longer delays, indicating that the response to long delays was less suppressed than the response to short delays. In other words, the strength of suppression increased over time during the stimulus presentation, in some neurons (Figure 5E, Wilcoxon signed rank test: p > 10 5). Note that the median delay did not change in 39% of the units indicating that collicular suppression did not change delay tuning in all collicular units.” (line 321-328)

Line 355 - sentence end is abrupt

Response: We modified the paragraph accordingly.

Line 367 - period

Response: changed

Line 392 - 'more strongly processed' - what does this mean? How was it quantified? How can you interpret this way based on what was quantified?

Response: “more strongly processed” is a vague and obviously a misleading expression. Here, we wanted to emphasize that the initial 450 ms of the response pattern to the multi-object sequence is predominantly determined by the presence of object A. After 450 ms, when object A is passed and echoes from object B are the first echoes arriving the bat's ears, object B mostly determine the response pattern to the multi-object sequence.

We rephrased the section as follow: “In summary, the initial 450 ms of the response pattern to the multi-object sequence is predominantly determined by echo information coming from object A. After passing object A, echoes from object B have the strongest influence on the response pattern to the multi-object sequence. Although collicular neurons responded to each echo of the multi-object sequence, collicular suppression ensures that echoes from the nearest object are mostly determining the response pattern to the multi-object sequence.” (line 392-397)

Discussion

Overall the discussion is highly 'bat-centric' and needs to argue why these findings could be of general interest.

Response: We rewrote the discussion section in a way to put our results into a broader scientific scope. For instance, we added a section about comparative results and discuss the relevance of our data with the results from other animal species.

Line 415-416 - Immunostaining is not going to provide the answer to the question posed.

Response: We deleted the section about frequency tuning in the IC (see comment above)

Line 423-425 - Does 2005 count as recent?

Response: We deleted the sentence due to rephrasing the entire discussion.

Line 425-427 - 'not surprising' - context is poorly established. The 2 bat species described in previous sentences are different. The present bat species is like one of them. So surprising or not depends on the comparison species - which should be done based on phylogeny.

Response: We rephrased the section without the expression “surprising”.

We wrote: “In P. parnellii, delay tuned neurons of the IC usually respond only to a combination of call and echo but not, or only sparsely, to call or echo alone (Yan and Suga, 1996; Macías et al., 2016). In contrast, collicular neurons of E. fuscus, another insectivorous species respond in 59% of the recordings to call and echo (Sanderson and Simmons, 2005). In C. perspicillata, the collicular units usually respond to call and echo which is indicated by two activity peaks per call-echo element in the median PSTHs (Figure 4D and 4E). Neuronal tuning to certain call-echo elements was only visible when integrating the number of spikes elicited by each call-echo element.” (line 501-507)

Line 429-430 - grammar

Response: fixed

Line 453 and 469- extra ')'

Response: We believe the extra “)” are actually correct, because two parentheses symbols were opened in that section.

Line 517 - 'of' instead of 'on'

Response: Changed

References

Simmons & Simmons 2011a and 2011b is the same paper; Kossl et al. 2014a and 2014b is the same paper.

Response: We apologize for that mistake and rechecked the reference list.
